# Spectrum of Hemoglobinopathies in Microcytic Hypochromic Anemia: A Cross-Sectional Study From Western India

**DOI:** 10.7759/cureus.109539

**Published:** 2026-05-24

**Authors:** Asha Goyal, Radha Sharma, Shiv Prakash Sharma, Yogesh K Gupta, Vivek Sharma

**Affiliations:** 1 Pathology, Rajasthan University of Health Sciences College of Medical Sciences, Jaipur, IND; 2 Preventive Medicine, Rajasthan University of Health Sciences College of Medical Sciences, Jaipur, IND

**Keywords:** beta thalassemia trait, hemoglobinopathies, high-performance liquid chromatography, india, microcytic hypochromic anemia

## Abstract

Introduction: Hemoglobinopathies are common genetic disorders with high prevalence in India. This study aimed to determine the spectrum of hemoglobinopathies among patients with microcytic hypochromic anemia at a tertiary care center in Jaipur, Western India.

Methods: This cross-sectional study enrolled 150 patients with microcytic hypochromic anemia (mean corpuscular volume (MCV) <80 fL, mean corpuscular hemoglobin (MCH) <27 pg) and clinically suspected hemoglobinopathy. Patients with a recent blood transfusion were excluded. Venous blood samples were analyzed using complete blood count and high-performance liquid chromatography (HPLC). Statistical analysis was performed using chi-square tests.

Results: Among 150 patients, 63 (42.0%) were diagnosed with the beta-thalassemia trait, making it the most common abnormality. A normal HPLC pattern was observed in 50 (33.3%) patients. Beta-thalassemia major was diagnosed in 12 (8.0%) patients, followed by hemoglobin S (HbS) trait in seven (4.7%), hemoglobin E (HbE) homozygous in six (4.0%), hereditary persistence of fetal hemoglobin (HPFH) in four (2.7%), and other rare variants in eight (5.3%) patients. Female predominance was observed among abnormal cases (62.0% females vs. 38.0% males; female: male ratio 1.6:1; χ²=5.76, p=0.016). The highest proportion of cases was observed in the <10 years age group (30.0%), followed by the 21-30 years age group (22.0%) (χ²=28.43, p=0.005).

Conclusion: Beta-thalassemia trait is the predominant hemoglobinopathy in this region. High prevalence in children under 10 years and females of reproductive age underscores the need for targeted screening programs in schools and antenatal clinics.

## Introduction

Hemoglobinopathies are inherited disorders characterized by structural abnormalities of hemoglobin or reduced synthesis of globin chains [1]. They represent the most common monogenic diseases worldwide, with an estimated 7% of the global population carrying an abnormal hemoglobin gene [2]. The World Health Organization estimates that approximately 300,000 babies are born annually with severe hemoglobin disorders, over 90% in low- or middle-income countries [3].

India bears a disproportionately high burden of hemoglobinopathies, with 3-4% of the population carrying beta-thalassemia (30-40 million carriers) [4]. Significant regional variations exist: hemoglobin S (HbS) is prevalent in tribal populations of Central, Western, and Southern India (carrier rates up to 48%), while hemoglobin E (HbE) is common in the Northeast (carrier rates up to 50%) [5,6].

Microcytic hypochromic anemia is a common presentation of thalassemia syndromes but is non-specific, as it may also occur in iron deficiency anemia. Therefore, accurate laboratory diagnosis using high-performance liquid chromatography (HPLC) is essential for management, genetic counseling, and prevention [7]. HPLC offers precise quantification of HbA2 and HbF, critical for diagnosing the beta-thalassemia trait [8].

Data on hemoglobinopathy patterns in Rajasthan remain limited. This study aimed to determine the prevalence and spectrum of hemoglobinopathies among patients with microcytic hypochromic anemia at a tertiary care center in Jaipur and analyze their distribution by age and gender.

## Materials and methods

Study design and setting

This was a cross-sectional, hospital-based observational study conducted in the Department of Pathology at Rajasthan University of Health Sciences College of Medical Sciences (RUHS-CMS) and its associated hospitals in Jaipur, Rajasthan, India. The study was carried out over six months, from August 2024 to February 2025.

Ethical approval

The study was approved by the RUHS-CMS Ethics Committee (approval number: RUHS-CMS/Ethics Comm./2024/338) on September 18, 2024. Written informed consent was obtained from all participants or their legal guardians (for minors). The consent form, available in English and Hindi, explained the study procedures, confidentiality, and voluntary participation.

Sample size calculation

The sample size was calculated assuming a hemoglobinopathy prevalence of 10% in India based on previous studies. The sample size was estimated using the formula $n = \frac{4pq}{d^2}$, where p=0.10, q=0.90, and d=0.05. The calculated sample size was 144. Rounding up, 150 patients were included to account for potential exclusions and incomplete data. 

Inclusion and exclusion criteria

Patients of all ages presenting with microcytic hypochromic anemia (mean corpuscular volume (MCV) <80 fL and mean corpuscular hemoglobin (MCH) <27 pg on complete blood count) with clinical suspicion of hemoglobinopathy based on family history, ethnic background, or characteristic clinical features were included in the study. This enriched sampling limits generalizability to the broader population. Children and parents of known patients with hemoglobinopathies and those willing to provide written informed consent were also included.

Patients with a history of blood transfusion within three months preceding sample collection (to avoid interference with HPLC results) and those unwilling to provide informed consent were excluded.

Data collection and sociodemographic variables

Demographic and clinical data were collected using a predesigned proforma, including age, gender, locality (rural/urban), caste, religion, education, socioeconomic status (modified Kuppuswamy scale), presenting symptoms (fatigue, weakness, dizziness, pallor, tachycardia, shortness of breath, easy bruising), and family history of hemoglobinopathies.

Laboratory methods

Sample Collection

Under aseptic conditions, 5 mL of venous blood was collected in ethylenediaminetetraacetic acid (EDTA) tubes (BD Vacutainer, Franklin Lakes, NJ, USA) and processed within two hours.

Complete Blood Count

CBC was performed using an automated hematology analyzer (Sysmex XT-4000i, Kobe, Japan). Parameters recorded included hemoglobin, RBC count, hematocrit, MCV, MCH, mean corpuscular hemoglobin concentration (MCHC), and red cell distribution width (RDW).

High-Performance Liquid Chromatography

Hemoglobin fraction analysis was performed using the Bio-Rad D10 HPLC system (Bio-Rad Laboratories, Hercules, CA, USA), which employs cation-exchange chromatography. Separation was based on the differential interaction of hemoglobin variants with the stationary phase. The system was calibrated, and quality control checks were performed daily. Each chromatogram quantified HbA, HbA2, HbF, and abnormal variants (HbS, HbE, HbD, etc.). Retention times and peak characteristics were used for variant identification per the manufacturer’s standards.

Diagnostic Criteria

Beta-thalassemia trait: HbA2 >3.9% with microcytic, hypochromic indices; beta-thalassemia major: HbF >70% with markedly reduced or absent HbA; HbS trait: presence of an HbS peak (typically 35-40%) in the S window; HbE homozygous: predominant HbE peak (>80%) with elevated HbF; and hereditary persistence of fetal hemoglobin (HPFH): elevated HbF (15-30%) without significant HbA2 elevation.

Statistical analysis

Data were entered into Microsoft Excel 2010 (Microsoft Corporation, Redmond, Washington, United States) and analyzed using IBM SPSS Statistics for Windows, Version 22 (Released 2013; IBM Corp., Armonk, New York, United States). Categorical variables were presented as frequencies and percentages (n (%)); continuous variables as mean ± standard deviation. The chi-square test was used to assess associations between categorical variables (age groups and hemoglobinopathy types; gender and abnormal patterns). A p-value <0.05 was considered statistically significant. Test statistic values (chi-square) are reported alongside p-values.

## Results

Study population characteristics

A total of 150 patients with microcytic hypochromic anemia and suspected hemoglobinopathy were enrolled. Age ranged from two months to 64 years (mean 26.4 ± 15.8 years). Of these, 82 (54.7%) were female and 68 (45.3%) male (female:male ratio 1.2:1).

Spectrum of hemoglobinopathies

HPLC revealed abnormal hemoglobin patterns in 100 (66.7%) patients. Beta-thalassemia trait was the most common abnormality (63 cases, 42.0%), followed by beta-thalassemia major (12, 8.0%), HbS trait (7, 4.7%), HbE homozygous (6, 4.0%), HPFH (4, 2.7%), and other rare variants (8, 5.3%) (Table 1).

**Table 1:** Distribution of hemoglobin patterns identified by HPLC (N=150) *Others include HbD Punjab, HbQ India, HbJ Meerut, and compound heterozygous states. HPLC: high-performance liquid chromatography; HbS: hemoglobin S; HbE: hemoglobin E; HPFH: hereditary persistence of fetal hemoglobin

Hemoglobin Pattern	Number of Cases (n)	Percentage (%)
Beta-Thalassemia Trait	63	42.0
Normal HPLC Pattern	50	33.3
Beta-Thalassemia Major	12	8.0
HbS Trait	7	4.7
HbE Homozygous	6	4.0
HPFH	4	2.7
Others (rare variants)*	8	5.3

Gender distribution of hemoglobinopathies 

Among 100 abnormal cases, females predominated (62, 62.0%) over males (38, 38.0%) (female: male ratio 1.6:1; χ²=5.76, p=0.016). Table 2 summarizes the gender distribution of specific hemoglobinopathies and all abnormal cases. A statistically significant female predominance was observed only for beta-thalassemia trait (63.5% female; χ²=4.59, p=0.032) and for all abnormal cases together (62.0% female; χ²=5.76, p=0.016). The other disorders did not show a significant gender difference.

**Table 2:** Gender distribution of specific hemoglobinopathies and all abnormal cases *Chi‑square test (df=1) for difference from expected 50:50 distribution. p <0.05 is considered statistically significant. Note: HPFH and other rare variants are not shown individually due to small numbers; they are included in “all abnormal cases”. HbS: hemoglobin S; HbE: hemoglobin E; HPFH: hereditary persistence of fetal hemoglobin

Hemoglobinopathy	Female n (%)	Male n (%)	Total N	χ² value	p-value*
Beta-Thalassemia Trait	40 (63.5)	23 (36.5)	63	4.59	0.032
Beta-Thalassemia Major	8 (66.7)	4 (33.3)	12	1.33	0.248
HbE Homozygous	4 (66.7)	2 (33.3)	6	0.67	0.414
HbS Trait	4 (57.1)	3 (42.9)	7	0.14	0.705
All Abnormal Cases	62 (62.0)	38 (38.0)	100	5.76	0.016

Age distribution

The age distribution of the study population and its association with specific hemoglobinopathies are presented in Table 3. Overall, the highest proportion of patients was in the <10-year age group (30.0%), followed by the 21-30-year age group (22.0%). Beta-thalassemia major was predominantly seen in children <10 years (10/12, 83.3%). Beta-thalassemia trait cases were most frequently observed in the <10 years and 21-30 years age groups. HbE homozygous and HbS trait were more frequent in the 21-30 years age group (reproductive age). The association between age group and type of hemoglobinopathy was statistically significant (χ²=28.43, p=0.005).

**Table 3:** Age distribution of study population and key hemoglobinopathies (N=150) *HbE homozygous + HbS trait combined (total 13 cases: six HbE + seven HbS). Statistical test: chi-square test for association between age groups and type of hemoglobinopathy (trait, major, HbE/HbS, others) - χ²=28.43, p=0.005. HbS: hemoglobin S; HbE: hemoglobin E

Age Group (Years)	Total Cases n (%)	Beta-Thalassemia Trait (n)	Beta-Thalassemia Major (n)	HbE/HbS Cases* (n)
<10	45 (30.0)	18	10	0
11–20	30 (20.0)	10	2	1
21–30	33 (22.0)	17	0	7
31–40	18 (12.0)	8	0	2
>40	24 (16.0)	10	0	3
Total	150 (100)	63	12	13

## Discussion

This study evaluated the spectrum of hemoglobinopathies among 150 patients with microcytic hypochromic anemia using HPLC. Approximately two-thirds of patients demonstrated abnormal hemoglobin patterns.

The high prevalence of beta-thalassemia trait (42.0%) observed in this study underscores the importance of screening strategies in India, as highlighted by Gorakshakar and Colah [9]. This prevalence is higher than that reported by Rao et al. (18.1%) [10] and Sachdev et al. (8.9%) [11], possibly reflecting differences in study populations and inclusion criteria. The prevalence of beta-thalassemia major (8.0%) is comparable to that of Bhokare et al. (9.6%) from Western India [12], possibly reflecting the referral-based nature of our tertiary care center.

Among structural variants, the HbS trait (4.7%) is higher than that of Rao et al. (2.8%) [10] but lower than Bhokare et al. (18.8%) [12], consistent with the known geographic variation of sickle cell disorders in India [13]. HbE homozygous (4.0%) is lower than South Indian reports (18.9%) [14], as expected given the Northeastern predominance of HbE [6].

The significant female predominance (62.0% of abnormal cases; p=0.016) may reflect healthcare utilization patterns among women of reproductive age, though this was not directly assessed, particularly in the context of antenatal screening [15].

The bimodal age distribution (peaks at <10 years and 21-30 years) has important public health implications. The higher frequency in children likely reflects the early presentation of severe disorders such as beta-thalassemia major (83.3% of cases in <10 years). The young adult peak coincides with reproductive age, highlighting opportunities for carrier screening and genetic counseling [16].

One-third of patients with microcytic hypochromic anemia had normal HPLC patterns, likely representing iron deficiency anemia, highly prevalent in the Indian population [17]. This underscores HPLC's role in distinguishing thalassemia carriers from iron deficiency for appropriate management [18].

Limitations

This single-center study with a moderate sample size (n=150) may introduce referral bias. Molecular confirmation was not performed for atypical cases. Patients with recent transfusions were excluded, potentially underrepresenting transfusion-dependent patients. Iron studies (serum ferritin, transferrin saturation) were not performed; hence, iron deficiency anemia could not be confirmed. Despite these limitations, the study provides valuable regional data from an understudied population in Western India.

## Conclusions

Beta-thalassemia trait is the predominant hemoglobinopathy among patients with microcytic hypochromic anemia in the Jaipur region (42.0%). The significant female predominance and bimodal age distribution (peaks in children <10 years and young adults of reproductive age) support targeted screening programs in schools and antenatal clinics. The high proportion of normal HPLC patterns highlights the importance of differentiating thalassemia carriers from iron deficiency anemia. These regional data provide a foundation for public health planning and routine HPLC-based screening in this population.
